# Delayed Sudden Respiratory Arrest After a High-energy Motorcycle Accident

**DOI:** 10.7759/cureus.6406

**Published:** 2019-12-17

**Authors:** Hiromichi Ohsaka, Kei Jitsuiki, Youichi Yanagawa

**Affiliations:** 1 Acute Critical Care Medicine, Juntendo University Shizuoka Hospital, Izunokuni, JPN

**Keywords:** respiratory arrest, traffic accident, vertebral artery, cerebellar infarction

## Abstract

A 50-year-old man driving a motorcycle at 100 kph crashed at a curve on a racing course. On arrival, he had clear consciousness, and his vital signs showed mild hypertension and tachycardia. His chief complaint was general pain. The only physiological finding was a labile injury. Whole-body computed tomography only showed fluid collection at the left maxillary sinus. While waiting on the results of a blood examination in the emergency room (ER), monitoring triggered an alarm due to a reduction in the percutaneous oxygen saturation. When a nurse checked him, he lost consciousness and entered respiratory arrest, showing left conjugated deviation and a palpable radial artery. He underwent indwelling tracheal intubation with mechanical ventilation. On the second hospital day, he regained consciousness and respiration and was therefore extubated. Brain magnetic resonance imaging revealed cerebellar infarction due to occlusion of a right vertebral artery, probably due to traumatic dissection. He was ultimately discharged on foot. This is a rare case of sudden-onset coma with respiratory arrest in the ER after a traffic accident due to occlusion of the right vertebral artery despite a clear consciousness on arrival. Physicians should closely monitor high-energy traffic accident victims, even when the patient has a clear consciousness and only minor physiological findings.

## Introduction

Missed injuries are a major concern in the management of trauma patients; in 25% of severely injured patients, at least one diagnosis remains undetected [1]. Traumatic blunt carotid or vertebral artery injuries are associated with mortality rates of up to 33% and a neurological morbidity of up to 38%; however, initially, a high proportion of cervical artery dissections remains asymptomatic [2].

We herein report a case of sudden respiratory arrest in the emergency room (ER) after a high-energy motorcycle accident due to occlusion of the vertebral artery in a patient who had a clear consciousness on arrival.

## Case presentation

A 50-year-old man driving a motorcycle at 100 kph crashed at a curve on a racing course. He was transported to our hospital by a physician-staffed helicopter. His only remarkable history was a duodenal ulcer. On arrival, he had a clear consciousness, a blood pressure of 140/88 mmHg, a heart rate of 103 beats per minute, a respiratory rate of 20/minute, a percutaneous saturation of 95% under room air, and an axillary temperature of 37.5°C. His chief complaint was general pain. The only physiological finding was a labile injury without any motor weakness at his extremities. He also did not demonstrate either hematoma or bruising in his neck. An ultrasound, roentgen of the chest and pelvis, and an electrocardiogram were all negative. Whole-body computed tomography (CT) only showed fluid collection at the left maxillary sinus. After suturing his labile wound, he waited on the results of a blood examination in the ER with monitoring. As he had complained of forehead pain and nausea, he received a drip infusion of acetaminophen. After 10 minutes, however, monitoring triggered an alarm due to a reduction in the percutaneous oxygen saturation. When a nurse checked him, he lost consciousness and entered respiratory arrest, showing left conjugated deviation and a palpable radial artery. He immediately underwent bag valve ventilation and indwelling tracheal intubation with mechanical ventilation after physicians arrived.

**Figure 1:**
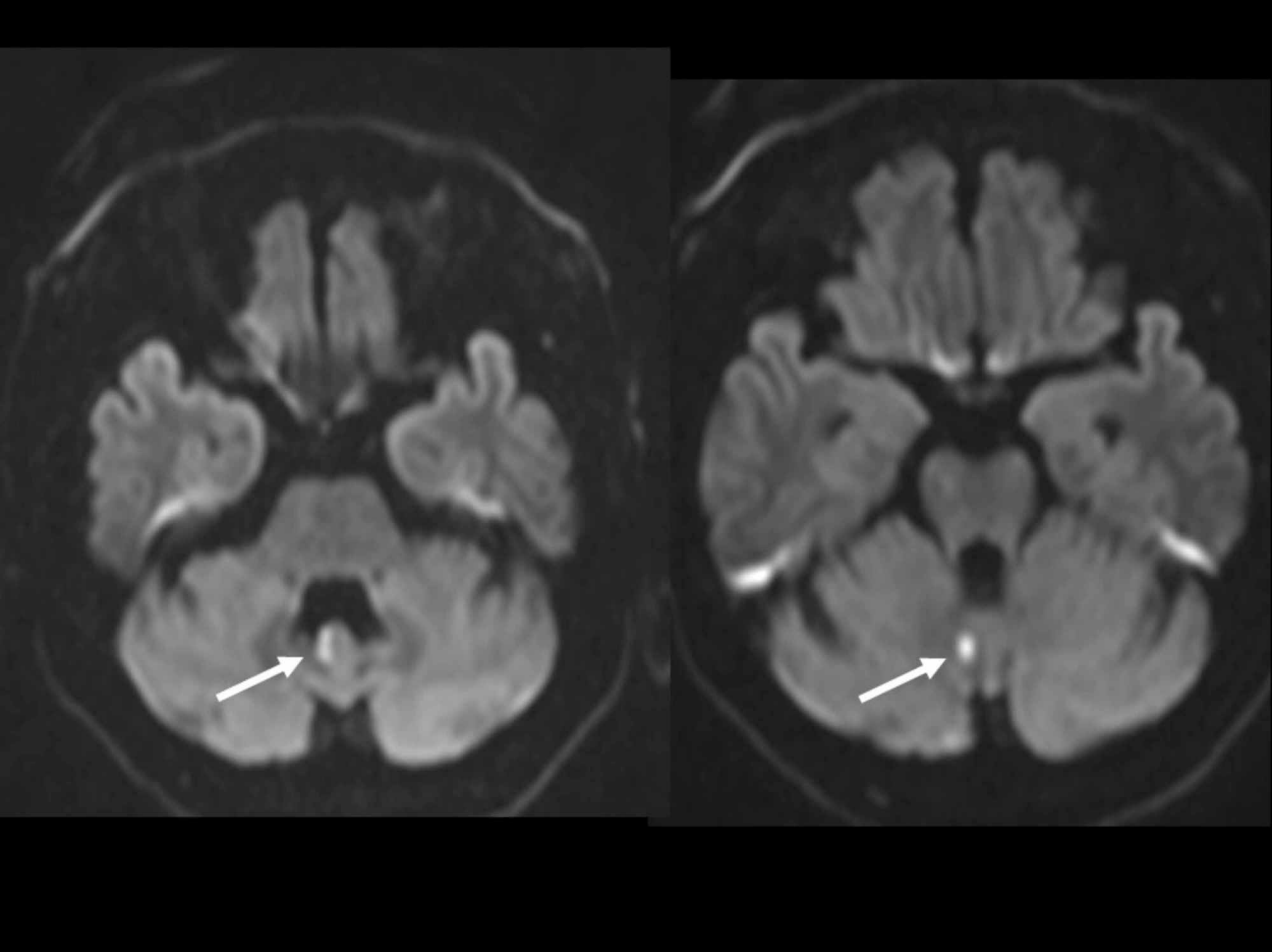
Diffusion-weighted scan of brain magnetic resonance imaging (MRI) on the second hospital day. MRI revealed cerebellar infarction (arrow).

**Figure 2:**
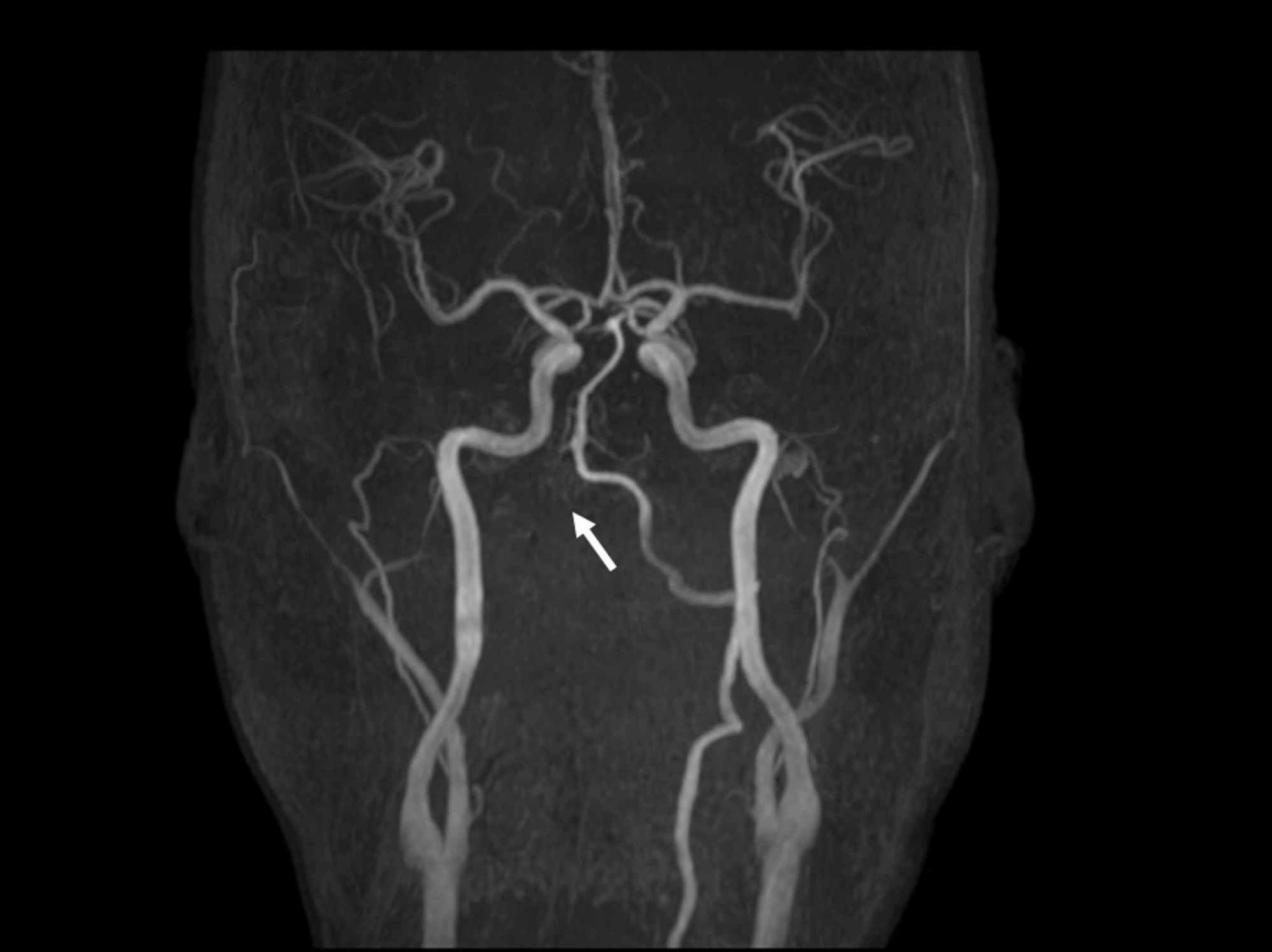
An image from brain magnetic resonance angiography (MRA) on the second hospital day. MRA revealed occlusion of the right vertebral artery (arrow).

**Figure 3:**
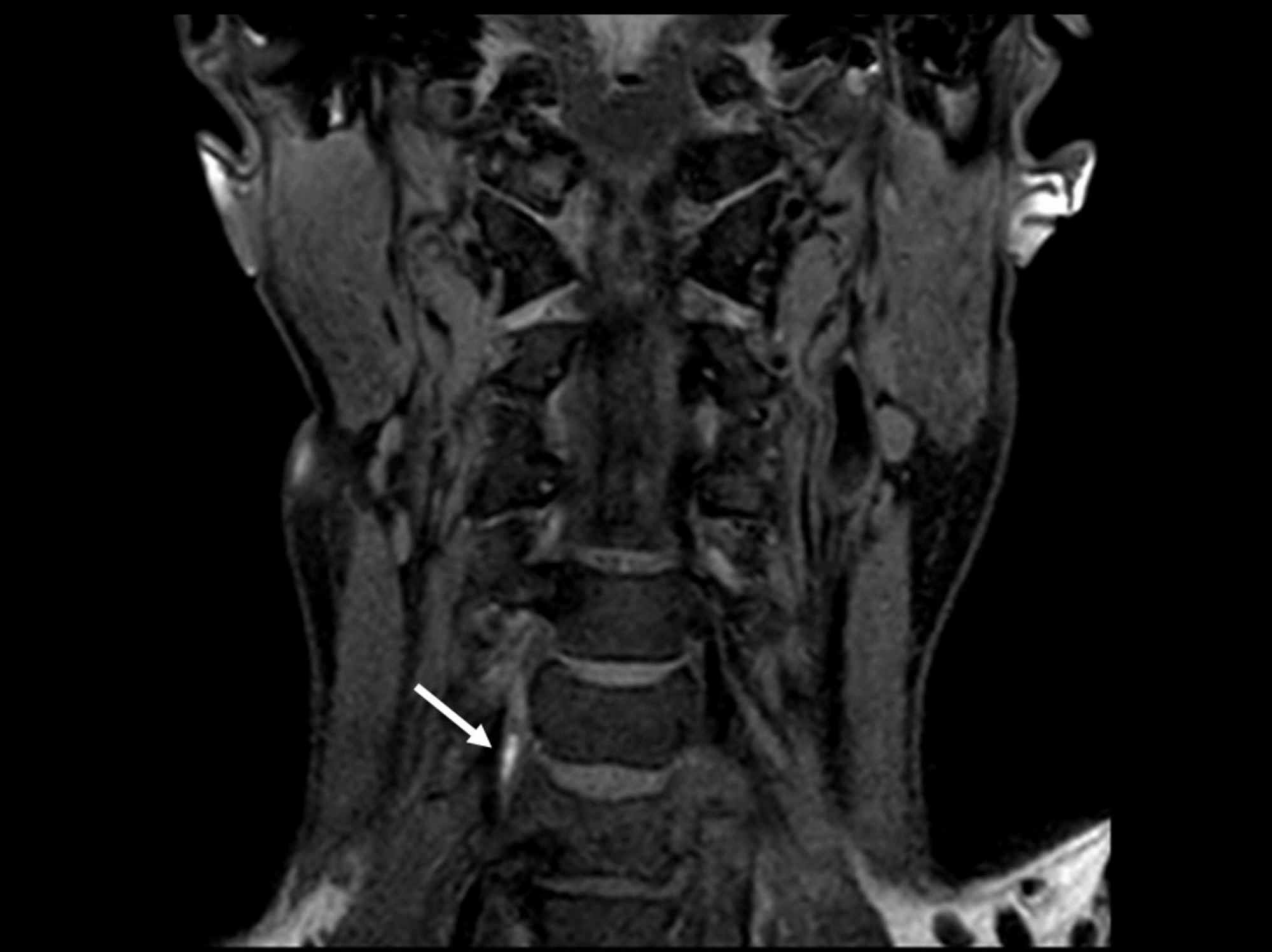
A coronal cervical T1-weighted image. The image shows a high-signal-intensity lesion (arrow) at the right vertebral artery, suggesting traumatic dissection.

A second head CT scan did not reveal any new traumatic lesions. He was moved to an intensive care unit with anticonvulsant. On the second hospital day, he regained consciousness and respiration and was therefore extubated. Brain magnetic resonance imaging revealed cerebellar infarction due to occlusion of a right vertebral artery, probably due to traumatic dissection (Figures 1-3).

Initially, he had an ataxic gate but became able to walk without any assistance with rehabilitation. After the confirmation of no recanalization of the right vertebral artery over seven days, he was discharged on foot and returned to his hometown.

## Discussion

This is a rare case of sudden-onset coma with respiratory arrest in the ER after a traffic accident due to occlusion of the right vertebral artery despite a clear consciousness on arrival.

Schicho et al. investigated 230 consecutive trauma patients with an injury severity score of ≥16 admitted to our level I trauma center using head-and-neck CT angiography [2]. Of these 230 patients, 6.5% had a cervical artery dissections. One death was caused by fatal cerebral ischemia. However, this case only had an injury severity score of 1. Chen reported a case of vertebral artery dissection probably caused by massage that almost resulted in the patient's death [3]. Even minor impact of the neck can injure the vertebral artery, although motor vehicle accidents or other impact mechanisms that induce rapid deceleration with stretching of the cervical artery over the lateral masses of the cervical vertebrae are the most common forms of trauma resulting in vascular dissections [4]. In addition to the initial signs and symptoms of trauma, neurological symptoms typically progress over the course of several hours to days, likely due to the propagation of a thrombus or distal embolization. As high-energy trauma has been shown to be an important contributor to mortality, physicians should monitor patients closely for the presence of undiscovered fatal injury, even those with a clear consciousness and minor physiological findings [5-7].

We did not perform MRI immediately after the patient’s condition worsened because we initially thought the present case complicated with tonic convulsion, and therefore performing MRI with mechanical ventilation was thus considered to be potentially dangerous if the patient’s vital signs suddenly became unstable [8]. While the present case fortunately showed a favorable outcome, however, he could have died due to brainstem infarction if he had undergone early intravascular intervention based on an early diagnosis using MR or CT angiography [9]. As there are no established guidelines concerning the treatment of traumatic vertebral artery injury, further studies should be carried out to identify the optimal treatment and diagnostic modalities for vertebral artery injury among traumatized patients without either head or neck injury.

## Conclusions

This is a rare case of sudden-onset coma with respiratory arrest in the ER after a traffic accident due to occlusion of the right vertebral artery despite a clear consciousness on arrival. Physicians should closely monitor high-energy traffic accident victims, even when the patient has a clear consciousness and only minor physiological finding.
